# Transport and stability of the vaccinia virus A34 protein is affected by the A33 protein

**DOI:** 10.1099/vir.0.049486-0

**Published:** 2013-04

**Authors:** Adrien Breiman, David C. J. Carpentier, Helen A. Ewles, Geoffrey L. Smith

**Affiliations:** Department of Virology, Faculty of Medicine, Imperial College London, Norfolk Place, London W2 1PG, UK

## Abstract

Vaccinia virus (VACV) has two infectious forms called intracellular mature virus and extracellular enveloped virus (EEV). Two of the seven viral proteins in the EEV outer envelope, A33 and A34, are type II membrane glycoproteins that each interact with another EEV protein called B5; however, evidence for direct A33–A34 interaction is lacking. The localization and stability of A34 is affected by B5 and here data are presented showing that A34 is also affected by A33. In the absence of A33, just as without B5, the level, localization and glycosylation profile of A34 was altered. However, the glycosylation profile of A34 without A33 is different to that observed in the absence of B5, and A34 accumulates in the Golgi apparatus rather than in the endoplasmic reticulum. Thus, A34 requires more than one other EEV protein for its processing and cellular transport.

*Vaccinia virus* (VACV) is the prototypical member of the genus *Orthopoxvirus* of the family *Poxviridae.* It replicates in the cytosol and produces multiple types of infectious virions (Smith *et al.*, 2002; Condit *et al.*, 2006; Roberts & Smith, 2008). The first infectious progeny is the intracellular mature virus (IMV), which is surrounded by a single lipid envelope (Dales & Siminovitch, 1961; Hollinshead *et al.*, 1999) and remains in the cell until cell lysis. However, some IMV are transported via microtubules to the early endosomes or *trans*-Golgi network where they are wrapped by two cellular membranes containing several VACV proteins. The resulting intracellular enveloped virus (IEV) is then transported on microtubules to the cell surface where the outer membrane fuses with the plasma membrane to externalise a double-enveloped virus by exocytosis. This virion is called cell-associated enveloped virus (CEV) if it remains on the cell surface, or extracellular enveloped virus (EEV) if it is released from the cell. The CEV/EEV outer membrane contains at least seven viral proteins: A33 (Roper *et al.*, 1996), A34 (Duncan & Smith, 1992), A56 (Shida, 1986), B5 (Engelstad *et al.*, 1992; Wolffe *et al.*, 1993), F13 (Blasco *et al.*, 1991), K2 (Turner & Moyer, 2006; Wagenaar & Moss, 2007) and VACV complement control protein (DeHaven *et al.*, 2010, 2011). These proteins are highly conserved between different orthopoxviruses (Gubser & Smith, 2002; Gubser *et al.*, 2004) and the loss of A33, A34, B5, A36 and F13 gives striking phenotypes *in vivo* (Smith *et al.*, 2002; Roberts & Smith, 2008). A34 is a type II transmembrane protein with different glycoforms between 23 and 28 kDa and its extracellular part contains a C-type lectin-like domain (Duncan & Smith, 1992). A K151D point mutation in the VACV strain Western Reserve (WR) A34, which is present naturally in the VACV International Health Department-J strain, causes an increase in EEV release (Blasco *et al.*, 1993). Similarly, deletion of the *A34R* gene (vΔA34R) from VACV WR caused a 25-fold increase in EEV, but such EEV had a fivefold reduction in specific infectivity (McIntosh & Smith, 1996). Deletion or suppression of the *A34R* gene caused a small plaque phenotype (Duncan & Smith, 1992; McIntosh & Smith, 1996), inability to form actin tails (Wolffe *et al.*, 1997; Sanderson *et al.*, 1998) and severe attenuation *in vivo* (McIntosh & Smith, 1996). A34 also affects the incorporation of other EEV proteins in the EEV outer membrane (Earley *et al.*, 2008; Perdiguero *et al.*, 2008; Roberts *et al.*, 2009).

The A33 protein is also a type II membrane protein with a C-type lectin-like fold with several glycoforms of 23–28 kDa (Roper *et al.*, 1996; Su *et al.*, 2010). In addition to *N*-glycosylations, A33 is also *O*-glycosylated (Payne, 1992), phosphorylated (Wolffe *et al.*, 2001) and acylated (Payne, 1992; Grosenbach *et al.*, 2000). Deletion of A33 leads to a reduction in plaque size (Roper *et al.*, 1998; Law *et al.*, 2002) and actin tail formation from the cell surface (Roper *et al.*, 1998) but a threefold increase in EEV release (Roper *et al.*, 1998). The A33 protein is also needed for the rapid spread of virus across cell monolayers by the repulsion of superinfecting EEV (Doceul *et al.*, 2010).

Both A34 (Röttger *et al.*, 1999; Earley *et al.*, 2008; Perdiguero *et al.*, 2008; Roberts *et al.*, 2009) and A33 (Perdiguero & Blasco, 2006) interact with the B5 protein, a 42 kDa type I glycoprotein also present in the EEV membrane (Engelstad *et al.*, 1992; Isaacs *et al.*, 1992). However, no direct A33–A34 interaction has been reported (Röttger *et al.*, 1999; Perdiguero *et al.*, 2008). Recently, we showed that in the absence of B5, A34 accumulated in the endoplasmic reticulum (ER), had an altered glycosylation and was degraded over time (Breiman & Smith, 2010). To investigate if other EEV proteins can affect A34, the expression level, glycosylation status and cellular localization of A34 was investigated in the presence and absence of A33.

RK13 cells were infected at 5 p.f.u. per cell for 16 h with VACV WR, vΔA33R or vΔA34R and cell lysates and extracellular virions (concentrated by centrifugation for 90 min at 12 000 ***g***) were analysed by immunoblotting using mouse mAb raised against the extracellular domains of A33 (33-1), A34 (34-1) (Breiman & Smith, 2010) and A36 (6.3) (van Eijl *et al.*, 2000), and a rat mAb against F13 (15B6) (Schmelz *et al.*, 1994) that was used as an infection and EEV loading control (Fig. 1a). A36 is a protein of the IEV envelope that stays in the plasma membrane during the exocytosis process and thus is not present in the EEV membrane (van Eijl *et al.*, 2000). As expected, A36 was detected in the cell lysates and only traces were visible in the EEV samples, showing that they are reasonably devoid of contaminating cellular material. In contrast, the F13 protein was present in both infected cells and EEV from all viruses (Fig. 1a). The level of A33 was reduced in vΔA34R EEV as noted previously (Perdiguero *et al.*, 2008) and A33 had an altered mobility on SDS-PAGE (Röttger *et al.*, 1999) (Fig. 1a). In addition, we noticed that the steady state level of A34 was reduced in both the vΔA33R EEV and infected cells lysates, suggesting that the absence of A33 affects A34 expression. To visualize the A33 and A34 migration profiles more clearly, lysates from infected RK13 cells were analysed by SDS-PAGE (15 % gel) in triplicate (one sample is shown in Fig. 1b) and the band intensities were quantified using a LI-COR Odyssey Quantitative Fluorescence Imaging System in conjunction with infrared fluorescent secondary antibodies (Fig. 1c). This confirmed that the absence of A33 affected the band pattern of A34. In addition, whereas the level of A33 was equivalent between vΔA34 and WR, the level of A34 was lower in vΔA33-infected samples compared with WR (Fig. 1d).

**Fig. 1.**
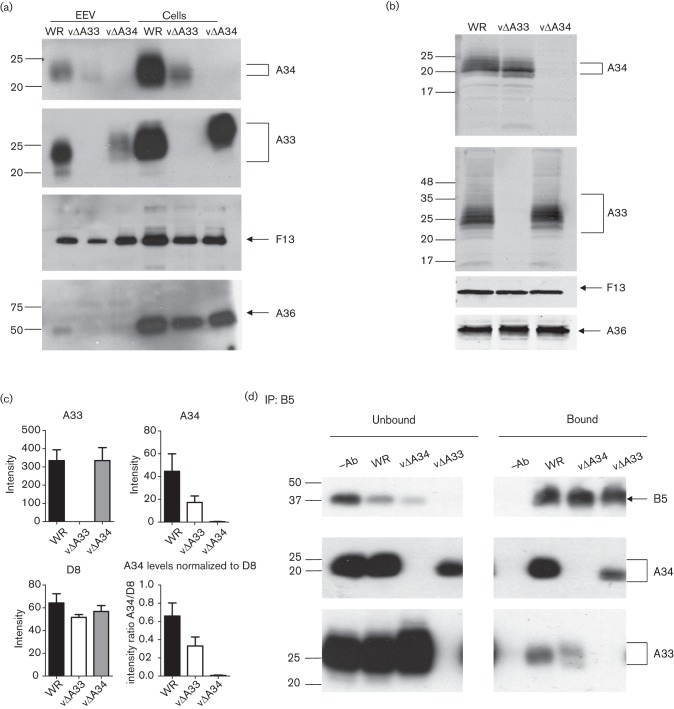
A34 and A33 interact with B5 and mutually affect each other. (a) RK13 cells were infected with WR, vΔA33R and vΔA34R for 16 h. Then cell lysates and EEV extracts were prepared and analysed by SDS-PAGE and immunoblotting with mAbs against A33, A34, A36 and F13 and detected by enhanced chemiluminescence. (b) Lysates from infected RK13 cells were prepared and analysed as in (a) but were imaged using LI-COR Odyssey Quantitative Fluorescence Imaging System to emphasize the banding pattern of the different glycoforms. (c) Band intensity of triplicate samples, prepared and analysed as in (b), was quantified using the LI-COR system to show relative protein levels at 16 h p.i. (d) RK13 cells were infected with WR, vΔA33R or vΔA34R at 5 p.f.u. per cell. Lysates were prepared at 16 h p.i. and subjected to immunoprecipitation by protein G beads chemically coupled to a rat mAb anti-B5. Pulled-down material and supernatants were analysed by SDS-PAGE (15 % gel) followed by immunoblotting with anti-A34, anti-A33 and anti-B5 mAbs. The ‘–Ab’ lane means that the lysates were incubated with beads only, without the B5 antibody. The positions of molecular mass markers are shown in kDa.

Next, we studied if A34 and A33 were interacting with B5 in an independent manner. For this, lysates of RK13 cells infected with WR, vΔA33R or vΔA34R as above were subjected to immunoprecipitation with protein G beads cross-linked with a rat mAb anti-B5 (19C2) (Schmelz *et al.*, 1994; Roberts *et al.*, 2009) and bound and unbound fractions were analysed by immunoblotting with a mouse mAb anti-B5 (36-6) (Breiman & Smith, 2010) and the 33-1 and 34-1 mAbs (Fig. 1d). As expected, both A34 and A33 were pulled-down with B5 in the WR lysates. In the vΔA34R lysates, A33 was still pulled-down with B5, although its migration profile was different from that seen with WR, as previously noted (Fig. 1a, b). In the absence of A33, the amount of A34 co-precipitated with B5 was markedly reduced. It was also reduced in the unbound fraction (Fig. 1d), indicating this reduction is probably due to a general decrease of A34 levels in the vΔA33R-infected cells, consistent with Fig. 1(a, b).

To investigate this further RK13 cells were infected at 5 p.f.u. per cell with WR or vΔA33R for 4, 8, 12 or 24 h and lysates were immunoblotted for B5, D8, A34 and A33 (Fig. 2a). In the vΔA33R lysates, A34 accumulated over time but at considerably reduced levels compared with those of WR at 12 and 24 h, while neither B5 nor D8 expression was affected. The pattern was different from that observed in the absence of B5, where there was a substantial expression of A34 up to 8 h post-infection (p.i.). followed by a rapid decrease (Breiman & Smith, 2010). However, as for vΔB5R, the A34 migration profile on SDS-PAGE changed to predominantly a faster migrating band in the vΔA33R cell lysates (Breiman & Smith, 2010) (Fig. 2a, b).

**Fig. 2.**
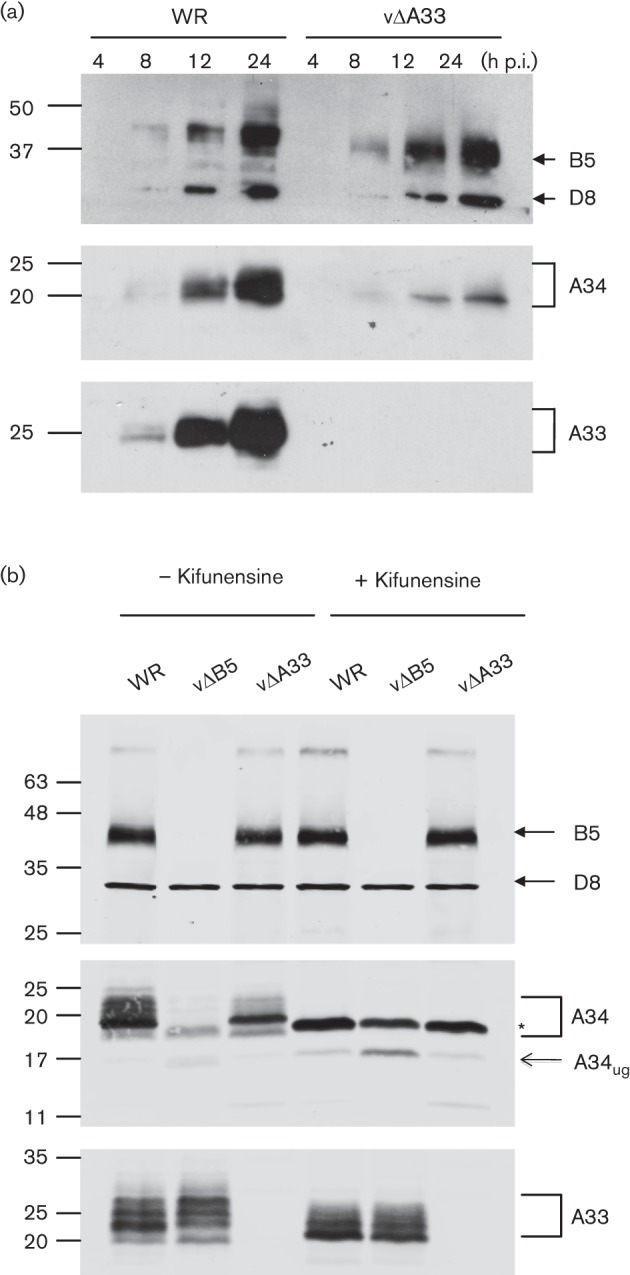
A34 protein levels and glycosylation are affected in the absence of A33. (a) RK13 cells were infected with WR or vΔA33R and harvested at the indicated time (h) p.i. Cell lysates were prepared and analysed by SDS-PAGE and immunoblotting with mAbs against A34, B5 and D8. The membrane was then stripped and reprobed with a mAb against A33. (b) RK13 cells were infected with WR, vΔB5R and vΔA33R at 5 p.f.u. per cell and incubated with or without kifunensine (20 µg ml^−1^). At 16 h p.i. cell lysates were prepared and analysed as in (a) and imaged using a LI-COR Odyssey Quantitative Fluorescence Imaging System. The asterisk indicates the A34 mannose-rich form (Man_9_ intermediate). A34_ug_ indicates the unglycosylated form of A34. The positions of molecular mass markers are shown in kDa.

To investigate if the A34 isoforms found in both deletion viruses were equivalent, we performed an overnight infection of RK13 with 5 p.f.u. per cell of WR, vΔA33R and vΔB5R in the presence or absence of the α-mannosidase I inhibitor kifunensine (Fig. 2b). As previously observed in the absence of B5 (Breiman & Smith, 2010), A34 migrated as a single band which looked indistinguishable from the band obtained with VACV WR, when trimming of mannoses was blocked by kifunensine. However, without kifunensine, the main A34 isoform in the vΔA33R-infected cells was about 22–23 kDa, cells were slightly larger than the band observed in vΔB5R and in the presence of kifunensine, although traces of other higher and lower bands were also apparent.

It was also noted that A33 had an altered migration in the absence of B5 with the appearance of higher bands than seen with WR. As that profile looks similar to the A33 profile in the absence of A34 (see Fig. 1), there is a possibility that this is linked to reduced A34 levels in vΔB5R, rather than a direct effect of the absence of B5. As A33 is essentially reduced to the same 19–20 kDa band by kifunensine treatment of either WR or vΔB5R, this change of A33 migration in the absence of B5 might be the consequence of alteration in *N*-glycosylation processing rather than to other modifications of A33.

Next, we investigated if the changes in A34 processing in the absence of A33 were associated with a change in A34 cellular localization. BSC-1 cells were infected with viruses at 2 p.f.u. per cell for 12 h, fixed with PBS–4 % paraformaldehyde (PFA) for 10 min on ice and then with PBS-8 % PFA for 20 min at room temperature. Fixed cells were permeabilized with 0.2 % Triton X-100 and incubated with anti-A34 mAb and a rabbit anti-protein disulphide isomerase (anti-PDI; Abcam) Ab to stain the endoplasmic reticulum or a rabbit anti-mannosidase II (anti-Man II; Abcam) to stain the Golgi apparatus (Fig. 3). In WR-infected cells, the anti-A34 mAb labelled the Golgi apparatus as well as punctate structures corresponding to virions in the periphery, as described previously (Lorenzo *et al.*, 2000), but no significant co-localization with PDI was observed. Some faint perinuclear staining lightly overlapping with Man II was also detected. In vΔA33R-infected cells, appreciably less A34 staining was observed on particles at the cell edges and A34 staining was predominantly intracellular. This largely did not co-localize with the PDI staining, but nicely overlapped with the Man II staining. Thus, in the absence of A33, A34 is localized mainly in the Golgi complex. This is different from that observed in vΔB5R-infected cells where A34 was retained in the ER (Breiman & Smith, 2010).

**Fig. 3.**
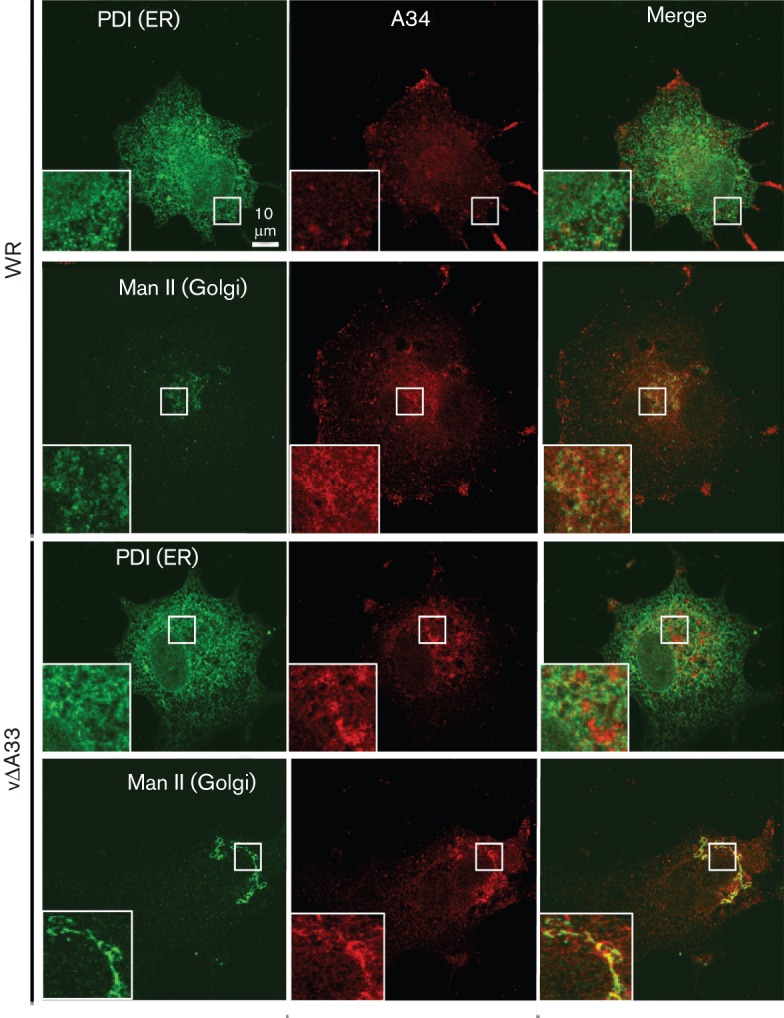
A34 accumulates in the Golgi in the absence of A33. BSC-1 cells were infected for 12 h with the viruses shown, fixed and processed for immunofluorescence using anti-A34 mAb followed by anti-mouse-Alexa 546 (red) and anti-PDI or anti-Man II followed by anti-rabbit-Alexa 488 (green). Samples were viewed on a Zeiss 510 Meta confocal microscope using Zeiss LSM software. The right panel of each row shows the merged image of the left and centre panel. Boxes within individual panels show regions of the cell before and after magnification. Bar, 10 µm.

In summary, data presented show that without the VACV protein A33, A34 protein levels are reduced, its glycosylation status is altered and it accumulates in the Golgi structures. Combined with data published about the dependency of A34 on B5 (Breiman & Smith, 2010), this shows that A34 requires at least two EEV proteins to be fully expressed, processed and transported to its final site of virion incorporation. It was also reported that B5 tended to accumulate in the Golgi in the absence of A33 (Chan & Ward, 2010). This is consistent with our findings because the B5–A34 interaction is well characterized. B5 also interacts with A33, and therefore it is possible that A33 primarily affects B5 localization and A34 localization is affected indirectly. Both vΔA34R- and vΔA33R-infected cells release greater amounts of EEV than WR-infected cells. However, the former releases about eight times more EEV than the latter. It is possible that the increased release of EEV by vΔA33R is linked to the reduced levels of A34 incorporation rather than to the absence of A33, but further investigations are needed to address this question.

Some of the results shown here and elsewhere also indicate that A33 itself is affected by the absence of B5 and/or A34 (Röttger *et al.*, 1999); however, the study of A33 is complicated by its multiple post-translational modifications. Overall, this study and others indicate that there are very complex interactions between these three EEV proteins, and this interdependency must be considered when studying EEV morphogenesis and re-entry processes.
